# Detection and Identification of *Bacillus cereus, Bacillus cytotoxicus, Bacillus thuringiensis, Bacillus mycoides* and *Bacillus weihenstephanensis* via Machine Learning Based FTIR Spectroscopy

**DOI:** 10.3389/fmicb.2019.00902

**Published:** 2019-04-26

**Authors:** Murat Bağcıoğlu, Martina Fricker, Sophia Johler, Monika Ehling-Schulz

**Affiliations:** ^1^Functional Microbiology, Institute of Microbiology, Department of Pathobiology, University of Veterinary Medicine Vienna, Vienna, Austria; ^2^Institute for Food Safety and Hygiene, Vetsuisse Faculty, University of Zurich, Zurich, Switzerland

**Keywords:** *Bacillus cereus*, FTIR spectroscopy, machine learning, artificial neural networks, diagnostics

## Abstract

The *Bacillus cereus* group comprises genetical closely related species with variable toxigenic characteristics. However, detection and differentiation of the *B. cereus* group species in routine diagnostics can be difficult, expensive and laborious since current species designation is linked to specific phenotypic characteristic or the presence of species-specific genes. Especially the differentiation of *Bacillus cereus* and *Bacillus thuringiensis*, the identification of psychrotolerant *Bacillus mycoides* and *Bacillus weihenstephanensis*, as well as the identification of emetic *B. cereus* and *Bacillus cytotoxicu*s, which are both producing highly potent toxins, is of high importance in food microbiology. Thus, we investigated the use of a machine learning approach, based on artificial neural network (ANN) assisted Fourier transform infrared (FTIR) spectroscopy, for discrimination of *B. cereus* group members. The deep learning tool box of Matlab was employed to construct a one-level ANN, allowing the discrimination of the aforementioned *B. cereus* group members. This model resulted in 100% correct identification for the training set and 99.5% correct identification overall. The established ANN was applied to investigate the composition of *B. cereus* group members in soil, as a natural habitat of *B. cereus*, and in food samples originating from foodborne outbreaks. These analyses revealed a high complexity of *B. cereus* group populations, not only in soil samples but also in the samples from the foodborne outbreaks, highlighting the importance of taking multiple isolates from samples implicated in food poisonings. Notable, in contrast to the soil samples, no bacteria belonging to the psychrotolerant *B. cereus* group members were detected in the food samples linked to foodborne outbreaks, while the overall abundancy of *B. thuringiensis* did not significantly differ between the sample categories. None of the isolates was classified as *B. cytotoxicus*, fostering the hypothesis that the latter species is linked to very specific ecological niches. Overall, our work shows that machine learning assisted (FTIR) spectroscopy is suitable for identification of *B. cereus* group members in routine diagnostics and outbreak investigations. In addition, it is a promising tool to explore the natural habitats of *B. cereus* group, such as soil.

## Introduction

*Bacillus cereus*, the name giving species of a group of genetically closely related species with highly variable pathogenic potential (Kolstø et al., 2009; Ehling-Schulz et al., 2011, 2019), is recognized as an important foodborne pathogen that provokes two different types of food poisoning – emesis and diarrhea (Hauge, 1955; Holmes et al., 1981). Two heat-labile tripartite protein toxin complexes, designated Nhe (non-haemolytic enterotoxin) and Hbl (haemolytic enterotoxin), have been linked to the diarrhoeal syndrome, while the emetic syndrome is caused by the depsipeptide toxin cereulide (Ehling-Schulz et al., 2004a; Stenfors Arnesen et al., 2008). Spore preparations of *B. thuringiensis* including its crystal toxin are commonly used biopesticides (Schnepf et al., 1998; Bravo et al., 2011) but *B. thuringiensis* has also been reported in the context of foodborne outbreaks and the presence of the aforementioned enterotoxin genes *nhe* and *hbl* as well as *in vitro* enterotoxicity has been described (Jackson et al., 1995; Gaviria Rivera et al., 2000; Johler et al., 2018). However, since in routine microbial diagnostics *B. cereus* and *B. thuringiensis* are not differentiated, the actual contribution of *B. thuringiensis* to foodborne outbreaks is hitherto unknown. More recently, a new *B. cereus* group species has been added, which is characterized by the production of a heat-labile necrotic protein enterotoxin, designated cytotoxin 1 (CytK1), and its thermotolerance (Guinebretiere et al., 2013). CytK1 has been first described in the context of a large foodborne outbreak in France, including three fatal cases (Lund et al., 2000).

In contrast to *B. cereus* and *B. thuringiensis*, which are rather ubiquitous and found in various food products, *B. cytotoxicus* seems to be associated with specific food products, such as mashed potato powder, potato pure and vegetable pure (Guinebretiere et al., 2013; Contzen et al., 2014; Heini et al., 2018). *B. cytotoxicu*s represents the thermotolerant member of the *B. cereus* group, (showing growth between 20°C and 50°C) while *B. weihenstephanensis* and *B. mycoides* are characterized by their high psychrotolerance, allowing them to grow at 5°C (Lechner et al., 1998; Guinebretiere et al., 2008). Although the latter two species can carry the enterotoxin genes *nhe* and *hbl*, they are mainly food spoiling microorganisms and their food poisoning potential seems to be rather low (Pruss et al., 1999; Ehling-Schulz et al., 2005; Hendriksen et al., 2006; Guinebretiere et al., 2008).

Despite several attempts have been made to differentiate the closely related *B. cereus* group members, an easy and cost-effective method for differential diagnostics is still lacking. Especially, the discrimination of *B. cereus* and *B. thuringiensis* in routine diagnostics is a challenge because both species are genetically intermingled (Guinebretiere et al., 2008; Ehling-Schulz et al., 2011; Zheng et al., 2017) and *B. thuringiensis* can only be differentiated from *B. cereus* by the presence of the *cry* genes. The *cry* genes are located on a megaplasmid and encode the crystal, insecticidal toxins, which delineated the species *B. thuringiensis* (Kronstad et al., 1983; Frederiksen et al., 2006). However, the high molecular polymorphisms of the *cry* genes, does not allow their reliable detection by standard PCR systems. In current food microbiology routine procedures, following the guidelines of the International Organization for Standardization (ISO), distinct species of the *B. cereus* are therefore not differentiated but subsumed as presumptive *B. cereus* for identification and enumeration in feed and food (ISO 7932:2004). However, since food safety authorities are aware of the limitations of these methods, initiatives have been started to include optional tests for differential diagnostics (see e.g., ISO 7932:2004/DAM 1:2018). In line with these initiatives, we assessed the potential of Fourier Transform Infrared (FTIR) spectroscopy for differentiation of *B. cereus* and *B. thuringiensis* as well as for the identification of the other *B. cereus* group species described above.

FTIR spectroscopy has already been applied successfully for the rapid identification of microorganisms (Helm et al., 1991; Wenning et al., 2008; Fricker et al., 2011) as well as for typing of *B. cereus* isolates (Ehling-Schulz et al., 2005). With this phenotypic method the entire chemical and biochemical composition of whole cells can be obtained by the interaction of mid-infrared light and the molecules present in the cells (Naumann et al., 1991), which yields species specific features. These “spectral fingerprints” are unique for each microorganism and allow the identification and / or differentiation at different taxonomic levels (Helm et al., 1991; Beattie et al., 1998; Rebuffo-Scheer et al., 2007; Wagener et al., 2014; Johler et al., 2016). Especially if FTIR spectroscopy is coupled to artificial intelligence (AI) systems, such as artificial neural networks (ANN), it is a powerful tool in microbial diagnostics (Bosch et al., 2008; Grunert et al., 2013; Schabauer et al., 2014; Lasch et al., 2018). ANNs are networks consisting of highly interconnected neural computing elements that acquire knowledge by a learning algorithm.

Because of the specific consumer risk related to *B. cereus* and *B. cytotoxicus*, the current lack of data about the role of *B. thuringiensis* in foodborne outbreaks as well as the specific spoilage potential of *B. mycoides* and *B. weihenstephanensis*, we aimed to establish a rapid diagnostic system with high throughput capabilities to discriminate the aforementioned species. To this end, we deployed ANN, as a machine learning method, to construct a classification system, based on bacterial FTIR spectral fingerprints. Further, the established method was applied for population analysis of *B. cereus* group isolates originating from two food poisoning outbreaks caused by emetic *B. cereus* and from soil samples of different geographic origins.

## Materials and Methods

### Bacterial Strains

A collection of 160 *Bacillus cereus* group strains, including 60 *B. cereus* (34 non-emetic *B. cereus*, 26 emetic *B. cereus*), 60 *B. thuringiensis*, 11 *B. cytotoxicus*, 14 *B. weihenstephanensis* and 15 *B. mycoides* isolates, was used for the training and the validation of the developed ANN model. Details on strains are provided elsewhere (Pruss et al., 1999; Ehling-Schulz et al., 2005; Carlin et al., 2006; Fricker et al., 2007; Guinebretiere et al., 2008; Heini et al., 2018; Johler et al., 2018) and in Supplementary Table S1 of the supplemental material. Strains were grown on plate count agar (5.0 g tryptone, 2.5 g yeast extract, 1.0 g D-glucose, 15 g agar per liter) at 30°C overnight. Strains were stored on plate count agar at 6°C for up to 3 weeks before they were transferred to fresh plates.

### Strains Characterization

All strains used in this study were either characterized in previous studies (see section “Bacterial Strains”), or additional tests were accomplished to assure the correct classification of the strains. The following parameters were used for strain characterization: starch hydrolysis, haemolysis, presence of the emetic toxin (*ces*) genes in *B. cereus*, the crystal toxin (*cry*) genes and / or the crystal toxin in *B. thuringiensis* and the cold shock protein A (*cspA*) gene in psychrotolerant strains as described (Bourque et al., 1993; Francis et al., 1998; Ehling-Schulz et al., 2004b, 2005; Hendriksen and Hansen, 2006). In addition, the pantothenate synthetase gene (*panC*) of selected strains was determined as described previously (Guinebretiere et al., 2008). Based on their *panC* sequences, strains were assigned to the phylogenetic groups described by Guinebretiere et al. (2008). The SplitsTreeTM software^1^ was used for SplitsTreeAnalysis of *panC* nucleotide sequences.

### Sample Preparation and Measurements of *B. cereus* Group Strains by FTIR Spectroscopy

The strains stored on plate count agar were streaked on tryptone soy agar (TSA) plates (Oxoid, Germany) and incubated at 25°C for 16–18 h. From this pre-culture the cells were grown as lawns on TSA plates at 25°C for 24 h ± 30 min. Samples were prepared as described previously (Fricker et al., 2011; Grunert et al., 2013). In brief, one loop (diameter 1 mm) of bacterial cells grown as confluent lawn was transferred into a 1.5 mL microcentrifuge tube containing 100 μL of sterile deionized water. The suspension was mixed by vortexing and aliquots containing 30 μl of the suspension were transferred onto a 96 well zinc selenite (ZnSe) optical plate (Bruker Optics GmbH, Ettlingen, Germany) and left for drying at 40°C for 40 min in order to create a thin, transparent film for FTIR measurements. The FTIR measurements were carried out using an IFS 28/B spectrometer and/or a HTS-XT microplate adapter coupled to a Tensor 27 FTIR spectrometer (Bruker Optics GmbH, Ettlingen, Germany) with spectral acquisition in transmission mode in the spectral range of 4000 to 500 cm^-1^ using the following parameters: 6 cm^-1^ spectral resolution, zero-filling factor 4, Blackmann-Harris 3-term apodization and 32 interferograms were averaged with background subtraction for each spectrum. The sample spectra were collected after background spectra were taken against an empty cell of the ZnSe plate. At least 10 independent measurements were prepared per strain to yield the number of spectra per strain required for the training and validation of the ANN (see section “Classification Model: Neural Network Architecture”).

### Spectral Data Analyses

The quality of FTIR spectral data was evaluated using OPUS software (version 7.8.5; Bruker Optics, Ettlingen, Germany), which assess the quality of spectra with respect to absorbance values, signal-to-noise ratio and intensity of the water vapor lines. On average, 90% of the spectra passed the quality test. Spectral pre-processing included the calculation of second derivatives using the Savitzky-Golay algorithm with 11 smoothing points and subsequent unit vector normalization using the OPUS software and the Orange data mining toolbox for Python (version 3.20.1) (Demsar et al., 2013) for plotting of processed data. The spectral windows from 3100 to 2800 cm^-1^ and 1800 to 700 cm^-1^ were pre-determined for further data processing and analysis.

Multivariate data analysis of FTIR spectra was performed using Unscrambler X (version 10.5, Camo AS, Norway) and MATLAB (Release 2018b, The MathWorks Inc., Natick, MA, United States) combined with the Deep Learning Toolbox running on a quad-core CPU laptop using single NVIDIA (Santa Clara, CA, United States) GeForce GTX 1060 (1152 CUDA cores) graphics card. Hierarchical cluster analysis (HCA) and principle component analysis (PCA) was used as an unsupervised method for spectral data analysis and ANN was employed as a supervised method to build up the classification model described in section “Classification Model: Neural Network Architecture.” In addition, HCA (using the Ward’s algorithm) of the processed spectra was performed to identify outlier spectra. For the establishment of the ANN, only spectra showing a heterogeneity < 1.2 were included. This procedure resulted in a total of 1307 spectra used for the establishment of the ANN (see section “Classification Model: Neural Network Architecture”).

### Classification Model: Neural Network Architecture

The Deep Learning Toolbox from MATLAB R2018b was applied to perform feature selection and to construct a neural network model. The pre-processed data set was divided into three subsets: training, validation and test set by setting randomly to 65, 20, and 15%, respectively. The training set employed for training the network comprised input and target vectors of the assigned set by computing the gradient and updating the network weights and biases. The validation set was used to validate that network by generalizing and allowing to stop training before overfitting by monitoring the error on the validation set during the training process. The test set was applied as an independent set to assess the network accuracy on data not being used in training or validation by measuring the suitability of the model on a real-world scenario.

### Statistics

The FTIR spectral data were evaluated using a normality test to decide whether the parametric or nonparametric statistical test should be used. For this purpose, the Mardia and Royston tests was applied (Mecklin and Mundfrom, 2004). These resulted in a non-normality assumption (the degree of significance, *p* < 0.0001), which is considered as monotonic but also nonlinear distribution, showing non-parametric statistic monotonic association between the variables of the spectral features. Further, since the data showed normal distribution, each *B. cereus* group member species were analyzed using the one-way ANOVA and Dunnett’s multiple comparison tests in GraphPad Prism 6 (GraphPad Software, Inc., United States) at which *p* < 0.05 was considered as statistically significant.

### Classification of *B. cereus* Group Isolates From Soil Samples and Isolates From Recent Food Poisonings by Emetic *B. cereus* With ANN Assisted FTIR Spectroscopy

Soil samples were collected in a field near Lausanne (Switzerland), Freising-Weihenstephan (Germany), Sarajevo (Bosnia and Herzegovina), and in Golden Bay (Malta). Ten g of each soil sample were diluted 1:10 in ¼ Ringer solution (Merck, Germany). Ten ml of the mixture were incubated at 80°C for 10 min and serial tenfold dilutions were plated on PEMBA (Oxoid, Germany) and plate count agar. *B. cereus* isolates were randomly selected and were subjected to further analysis. Furthermore, two food samples, a rice dish implicated in a foodborne outbreak in a day care center for children in Southern Germany (Fricker et al., 2007), and a milk rice, implicated in a foodborne outbreak in Northern Germany (Wambo et al., 2011), have been included. A total of 25 g of sample was homogenized in 225 ml BHIG broth (Brain Heart Broth (Merck, Germany) supplemented with 0.1 % Glucose) and serial tenfold dilutions were plated as described for the soil samples. Isolates were measured by FTIR as described above and were identified by ANN assisted FTIR spectroscopy.

## Results

### Analysis of FTIR Spectra

**FIGURE 1:**
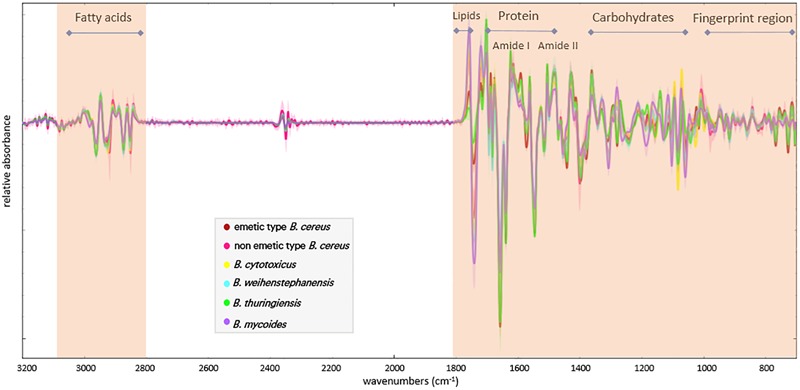
Representative processed (second derivative and unit vector normalized) FTIR spectra of the *B. cereus* group members, which were used in the ANN model for classification. The selected wavenumbers ranges are highlighted. Bars indicate spectral regions, which have been assigned to different microbial components [according to (Helm et al., 1991; Maquelin et al., 2002)].

Fourier Transform Infrared spectra from all strains included in this study (see Supplementary Table S1) were recorded and the quality of spectra was verified by the quality test provided with the OPUS software. Only FTIR spectra passing this test were used for further analysis. Second derivatives of FTIR spectra were calculated and unit vector normalized as described in material and methods. Representative processed spectra from the different *B. cereus* group species are presented in Figure 1. Next, processed spectra were analyzed by principle component analysis (PCA) as an unsupervised method to gain an overview on the distribution of species and strains. However, the score plots of the PCA analysis of FTIR spectra from the different *B. cereus* group members did not show a clear species-specific grouping (Supplementary Figure S1). Similar to the metabolic fingerprinting of *B. cereus* group strains by FTIR, SplitsTree analysis of *panC* sequences resulted in mixed species clusters. In particular, *B. cereus* and *B. thuringiensis* are intermingled and scattered over different clusters (Supplementary Figure S2).

Since unsupervised methods, such as PCA, did not allow to discriminate the *B. cereus* group members based on their FTIR spectral fingerprints, the suitability of supervised systems for species discrimination was explored. To this end, an ANN was constructed as outlined below, using the spectral wave number ranges indicated in Figure 1.

### Construction of the ANN Model

**FIGURE 2:**
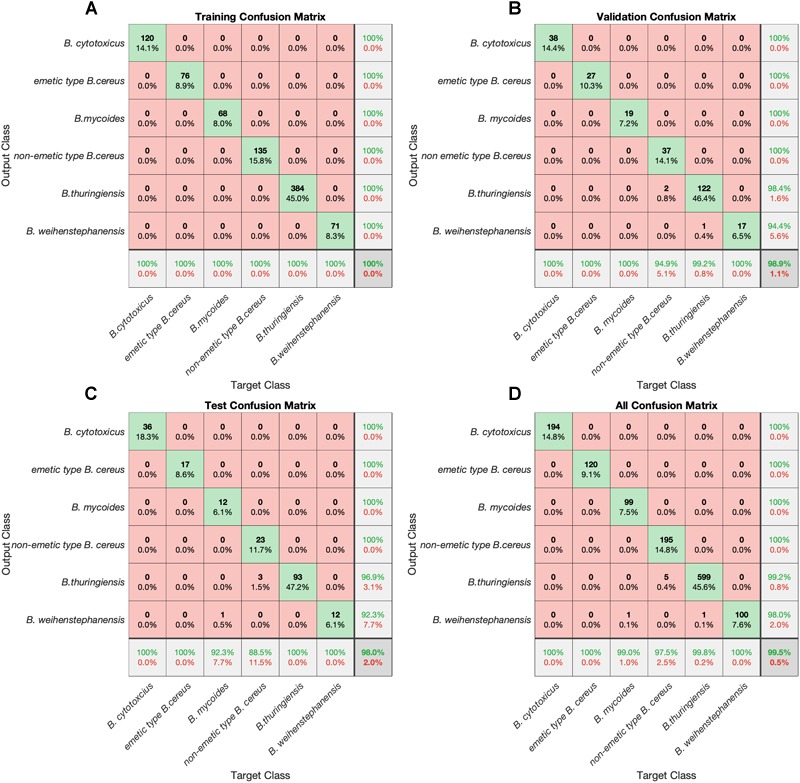
Confusion matrices. Overall percentages of correct and incorrect classifications. Correct classifications are the green squares on the matrix diagonal. The red squares represent incorrect classifications. The number of the spectra and the portion of spectra are given in each cell box. If the network is accurate, then the percentages in the red squares are small which indicates few misclassifications. Confusion matrices for each sets were shown as following **(A)** training set **(B)** validation set **(C)** test set and **(D)** all confusion matrices in one matrix.

The network model constructed was a two-layer feed-forward network with a sigmoid transfer function in the hidden layer, and a softmax transfer function in the output layer. For training multilayer feed-forward network, scaled conjugate gradient numerical optimization algorithm was employed to optimize the performance functions. Roughly, the network weights and biases alternates into such a way that the performance function decreases most rapidly, iterating until the negative of the gradient converges (Hagan et al., 1996). Once training the network has been completed, its performance was evaluated by using cross-entropy and percent misclassification error as well as by analyzing the results using visualization tools, such as confusion matrices (Figure 2). The confusion matrix helps to evaluate the model by looking at the percentages of correct and incorrect classifications. Correct classifications are presented on the green squares on the matrix diagonal. The red squares represent incorrect classifications. If the network is accurate, then the percentages in the red squares are small which indicates the few misclassifications.

**FIGURE 3:**
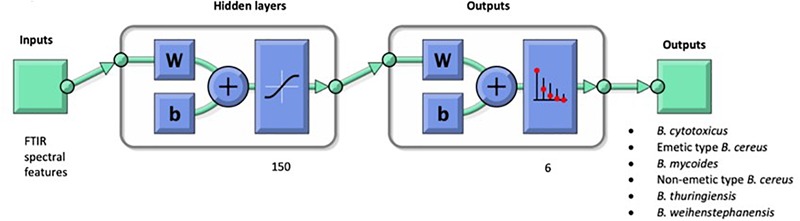
Architecture of the ANN model constructed from FTIR spectra of *B. cereus* group member species. The model outcomes the *B. cereus* group in six different subgroups for the discrimination of non-emetic type *B. cereus*, emetic type *B. cereus, B. thuringiensis* and *B. weihenstephanensis, B. mycoides, B. cytotoxicus*. W and b refer to the network’s adjustable parameters which are weight matrices and bias vectors. Once the network is trained, its bias and weight values formed into a vector. The single vector is then redivided into the original biases and weights.

In order to improve the generalization in the neural network model, a regularization method was employed. The regularization involves modifying the performance function, which is normally chosen to be the sum of squares of the network errors on the training set. In addition, the network has been trained 100 times. By calculating the mean squared error for the average output, which was lower than most of the individual performances, the outputs for any input were averaged. The constructed ANN model is depicted in Figure 3. Further, receiver operating characteristics (ROC) curve analysis was performed in order to evaluate the predictive ability of the established model. ROC curve, the true positive rate (i.e., sensitivity) versus false positive rate (i.e., 1-specificity), was plotted using the statistical data obtained by ANN classification based on FTIR spectroscopic data. The area under the ROC curve (AUC) is a measure of classification model performance that indicates a successful classification model, if it is close to 1. Thus, a good model accuracy can be assumed for our established ANN as the AUC values for each species are close to 1 (see Supplementary Figure S3).

### Results of the ANN Model

The architecture of ANN model was designed in such a way that in a one level net, the spectrum of an unknown *B. cereus* group isolate is assigned to one of the groups specified as (1) emetic *B. cereus*, (2) non-emetic *B. cereus*, (3) *B. thuringiensis*, (4) *B. weihenstephanensis*, (5) *B. mycoides* and (6) *B. cytotoxicus*.

Strains of the *B. cereus* group used for training, listed in Supplementary Table S1 of the supplemental material, could be unequivocal assigned to one of the specific *B. cereus* group members mentioned above in the architecture of the model. The ANN model, which was trained with randomly assigned spectra of 65% of the processed FTIR spectral dataset strains, yielded 100% correct identification (Figure 2). Further, in the validation of the trained model with randomly assigned 20% of the pre-processed FTIR dataset, only two spectra from non-emetic *B. cereus* and one from *B. thuringiensis* isolates were misidentified, whereas all spectra from emetic type of *B. cereus*, from *B. weihenstephanensis*, from *B. mycoides* and from *B. cytotoxicus* were identified correctly. The independent test set, consisting of randomly assigned 15 % of the dataset, which were unknown to the ANN model, achieved a correct identification rate of 98.0% (Figure 2). Overall, the constructed ANN model yielded 99.5 % correct classification. It was able to identify all spectra of emetic type of *B. cereus, B. cytotoxicus* and *B. weihenstephanensis* strains (100%) correctly, 99.0% of the *B. mycoides*, 97.5% of the non-emetic type *B. cereus*, 99.8% of the *B. thuringiensis* spectra.

### Application of the Developed ANN Assisted FTIR Spectroscopy for the Classification of *B. cereus* Group Isolates From Different Soil Samples and Rice Dishes Associated With Emetic Foodborne Outbreaks

After establishment of the ANN model, it was used to analyze *B. cereus* populations in soil samples of different geographical origin and in food samples, collected in frame of two foodborne outbreaks in Germany. The soil samples were collected near Freising-Weihenstephan (Germany), Golden Bay (Malta), Lausanne (Switzerland), and Sarajevo (Bosnia and Herzegovina). The rice dish was sampled in a day care center in Southern Germany where 17 children started vomiting after consumption of the reheated meal. The milk rice sample originates from a foodborne outbreak in Northern Germany, which effected 43 children from different kindergartens and 3 adults. From each sample, 66 to 110 *B. cereus* group isolates were obtained and subjected to FTIR spectroscopy. Subsequent chemometric analysis was carried out using the novel ANN. The main population components varied between the different samples (Table 1).

**Table 1:** Classification of soil sample isolates from different origins and from food poisoning cases via FTIR spectroscopy with the developed ANN model.

	Golden Bay, Malta	Field near Lausanne, Switzerland	Field near Weihenstephan, Germany	Sarajevo, Bosnia and Herzegovina	Rice dish from a food poisoning	Milk rice from a food poisoning
Non-Emetic *B. cereus*	27 (24.5%)	29 (31.9%)	39 (38.2%)	58 (71.6%)	19 (28.8%)	61 (59.8%)
Emetic *B. cereus*	0	2 (2.2%)	3 (2.9%)	0	45 (68.2%)	32 (31.4%)
*B. thuringiensis*	13 (11.8%)	7 (7.7%)	7 (6.9%)	3 (3.7%)	2 (3.0%)	9 (8.8%)
*B. weihenstephanensis*	0	19 (20.9%)	31 (30.4%)	5 (6.2%)	0	0
*B. mycoides*	70 (63.6%)	34 (37.9%)	22 (21.6%)	15 (18.5%)	0	0
*B. cytotoxicus*	0	0	0	0	0	0
*Total no. of isolates*	110 (100%)	*88 (100%)*	100 (100%)	*81 (100%)*	*66 (100%)*	102 (100%)

*Percentages of the isolates per group were given in parenthesis.*

The main part of the Golden Bay soil isolates (64 %) was classified as *B. mycoides*, while *B. cereus* was the predominant species in the soil sample from Sarajevo (72%). The majority of the isolates from the soil samples from the area of Lausanne were classified as *B. mycoides* and *B. cereus*, (38 and 32%, respectively), while in the soil samples from Weihenstephan *B. cereus* and *B. weihenstephanensis* showed the highest prevalence (38 and 30%, respectively). As expected, the majority of isolates (68%) from the rice dish connected to the emetic food poisoning in Southern Germany belonged to the emetic type of *B. cereus*. However, unexpectedly, non-emetic *B. cereus* isolates represented the majority of isolates (60%) from the emetic foodborne in Northern Germany while only 31% of isolates in the latter sample belong to emetic *B. cereus* (Table 1). *B. thuringiensis* was detected in all samples in low abundancies, ranging from 3 to 12%. *B. cytotoxicus* was not detected in any of the samples.

## Discussion

The discrimination between the members of the *B. cereus* group in food microbiology as well as in clinical diagnostics is primarily based on distinct pathogenic characteristics, such as toxin formation, and phenotypical characteristics, such as the formation of crystalline inclusion bodies for *B. thuringiensis*, the ability to grow at low temperature for *B. weihenstephanensis* or the rhizoid colony morphology of *B. mycoides*. Because of the close genetic relationship among members of the *B. cereus* group and the fact that species are phylogenetically not clearly separated (Guinebretiere et al., 2008; Kolstø et al., 2009) (see also Supplementary Figure S2), it was proposed to regard them as one species (Carlson et al., 1994; Helgason et al., 2000; Rasko et al., 2005). More recently, it has also been suggested to split them up in several genomospecies, based on average nucleotide identity (ANI) values (Liu et al., 2017). However, both concepts would cause problems for diagnostics and assignment to distinct biological risk groups (Priest et al., 2004; Ehling-Schulz et al., 2019). Thus, we assessed the potential of FTIR spectroscopy in combination with machine-based learning to establish a classification system for the *B. cereus* group members *B. cereus* (non-emetic and emetic type), *B. thuringiensis, B. weihenstephanensis, B. mycoides, B. cytotoxicus*, which are of major importance in food microbiology.

The implementation of ANNs in combination with FTIR spectroscopy has been reported to allow a more accurate differentiation of closely related species and strains than unsupervised methods, such as hierarchical cluster analysis (HCA) and principal component analysis (PCA) (Rebuffo et al., 2006; Grunert et al., 2013). These findings are corroborated by our current study. Our work provided evidence that the application of a machine learning model with the aid of ANN is a powerful tool for classification of bacterial species and subtypes (that are difficult to discriminate in routine diagnostics) based on their FTIR spectral fingerprints. ANNs have the advantage that the most discriminative wave numbers between species or isolates are weighted according to their importance for the differentiation (Schmitt and Udelhoven, 2001), while, by contrast, only windows between certain wave numbers can be selected for HCA. The trained ANN model in our study reached an overall correct identification rate of 100% in the internal and 98.0% in the external validation. These results demonstrate the high performance of ANN assisted FTIR spectroscopy for the differentiation of the closely related *B. cereus* group members.

Next, we applied the established ANN assisted FTIR spectroscopy method to gain insights into the population structure of soil samples from different geographical origins and food samples from outbreaks caused by emetic *B. cereus* in Germany. The classification results, presented in Table 1, revealed significant population differences in such kind of samples. The psychrotolerant members of the *B. cereus* group, *B. mycoides* and *B. weihenstephanensis* were predominant in the soil samples from Germany, Malta, Switzerland (53 to 70% per sample) but were not found in the sample from foodborne outbreaks. Only few of the soil isolates were classified as *B. thuringiensis* (3 to 12% per sample). Similar observations have been reported from a study of sandy loam in Denmark. The latter type of soil contained mainly *B. weihenstephanensis* and *B. mycoides* (94%) but was free of *B. thuringiensis* and only 6% of the population represented *B. cereus* isolates (Hendriksen et al., 2006). The differences in populations of soil samples concerning mesophilic and psychrotolerant *B. cereus* group members might be, at least partially, explained by the annual average temperature at the different sampling sites (von Stetten et al., 1999). The low number of soil isolates classified to the emetic type of *B. cereus* corroborates previous findings and suggest that emetic strains occupy specific, still largely unknown natural niches (Altayar and Sutherland, 2006; Ehling-Schulz et al., 2015).

In contrast to soil samples, none of the isolates from the foodborne outbreaks was classified to the psychrotolerant members of the *B. cereus* group (Table 1), fostering the hypothesis that the psychrotolerant members of the *B. cereus* group are rather a food spoilage than a food safety problem (Ehling-Schulz et al., 2005; Guinebretiere et al., 2008). However, apart from the isolates assigned to the emetic type of *B. cereus* (68 and 32%), a considerable number of isolates was classified as non-emetic *B. cereus*. In one emetic outbreak, the number of non-emetic isolates (60%) even exceeded the number of emetic isolates (31%). Mixed populations of *B. cereus* group isolates in such samples have also been reported from other outbreaks (Pirhonen et al., 2005; McIntyre et al., 2008; Schmid et al., 2016), strengthening the demand to analyze more than one colony from food samples implicated in food poisonings. For instance, using a WSG approach for the investigation of a recent *B. cereus* outbreak in upstate New York, emetic as well as non-emetic *B. cereus* isolates were identified in human and food samples connected to the outbreak (Carroll et al., 2019), corroborating the results from the analysis of two outbreaks in Germany by our newly established ANN assisted FTIR method. However, the in-depth investigation of a *B. cereus* outbreak by WGS recently presented by Carroll et al. also pinpoints the current bioinformatic challenges related to such investigations, which is still a drawback for the implementation of WGS in routine diagnostics. By contrast, our ANN assisted FTIR does not require specialized bioinformatic expertise, which facilitates its implementation in routine diagnostics.

Nevertheless, results from both, the WGS approach used by Carroll et al. (2019) as well as from our ANN assisted FTIR method, highlight the importance of accompanying molecular and biochemical analyses for detection of the pathogen and /or its toxin directly in the food, especially in outbreak situations (Fricker et al., 2007; Bauer et al., 2010). Notable, none of the isolates from the soil nor from the foodborne outbreak samples from Germany (see Table 1) or New York upstate (Carroll et al., 2019) was classified as *B. cytotoxicus*, which strengthen the hypothesis that *B. cytotoxicus* is rather rare in nature and restricted to very specific, yet to explore, niches (Contzen et al., 2014; Heini et al., 2018).

## Conclusion

In summary, a machine learning approach was successfully employed to establish an ANN assisted FTIR spectroscopy method for the rapid and cost-efficient differentiation of closely related *B. cereus* group members, such as *B. cereus* and *B. thuringiensis*. This novel method was applied to classify *B. cereus* group members in different soils and food samples implicated in food poisoning. Our work demonstrates the high complexity of natural *B. cereus* group populations, which is a challenge for routine diagnostics as well as for investigations in outbreak situations. The developed method is a promising tool for investigation of *B. cereus* group populations in different kinds of samples requiring high throughput capacities, such as soil, water or food habitats. It could help not only to elucidate the so far largely unknown role of *B. thuringiensis* in foodborne outbreaks but also to decipher the rather unexplored natural habitats of the highly potent toxin producers, *B. cytotoxicus* and emetic *B. cereus*.

## Author Contributions

ME-S and MF conceived and designed the study. MF performed the experiments. MB and MF carried out the chemometric analyses. SJ contributed strains and carried out genotypic analyses. MB, MF, and ME-S wrote the manuscript. ME-S acted as overall study coordinator. All authors revised the manuscript.

## Conflict of Interest Statement

The authors declare that the research was conducted in the absence of any commercial or financial relationships that could be construed as a potential conflict of interest.

## Footnotes

1 http://www.splitstree.org
