# Supplementary material for: Detection and Identification of Bacillus cereus, Bacillus cytotoxicus, Bacillus thuringiensis, Bacillus mycoides and Bacillus weihenstephanensis via Machine Learning Based FTIR Spectroscopy
Source: Front Microbiol. 2019 Apr 26;10:902. doi: 10.3389/fmicb.2019.00902 (PMC6498184; doi:10.3389/fmicb.2019.00902)
Supplement: Supplementary file 1 [file Data_Sheet_1.PDF]

Supplementary information

Table S1. Strain list used for the construction of the artificial neural network. The list contains the training, validation and independent test set strains for the *B. cereus* group.

**species**	**codes**	**information**
B. cereus (emetic)	F2938/03	clinical isolate
B. cereus (emetic)	F3351/87	clinical isolate
B. cereus (emetic)	F3876/87	clinical isolate
B. cereus (emetic)	F47/94	food borne outbreak
B. cereus (emetic)	F4810/72 (SMR-178)	food borne outbreak
B. cereus (emetic)	IH41385	clinical isolate
B. cereus (emetic)	MHI 135	food
B. cereus (emetic)	MHI 1471	food borne outbreak
B. cereus (emetic)	MHI 1631	food borne outbreak
B. cereus (emetic)	MHI 1745	food borne outbreak
B. cereus (emetic)	MHI 87	food isolate
B. cereus (emetic)	NC 7401	food borne outbreak
B. cereus (emetic)	RIVM 124	clinical isolate
B. cereus (emetic)	RIVM 379	food
B. cereus (emetic)	RIVM BC51	food borne outbreak
B. cereus (emetic)	SDA A 116	food
B. cereus (emetic)	SDA GR 177	food + environment
B. cereus (emetic)	UHDAM 1IFI (1)	food
B. cereus (emetic)	UHDAM 1IFI (3)	food
B. cereus (emetic)	UHDAM 3/pkl	clinical isolate
B. cereus (emetic)	UHDAM B315	food
B. cereus (emetic)	UHDAM ML127	food borne outbreak
B. cereus (emetic)	WSBC 10881	food borne outbreak
B. cereus (emetic)	WSBC 10895	food
B. cereus (emetic)	WSBC 10898	food
B. cereus (emetic)	WSBC 10900	food
B. cereus (non emetic)	3571 (M12)	food
B. cereus (non emetic)	ATCC 10876	environment
B. cereus (non emetic)	ATCC 10987	food
B. cereus (non emetic)	ATCC 11950	unknown
B. cereus (non emetic)	ATCC 27877	environment
B. cereus (non emetic)	ATCC 7064	clinical isolate
B. cereus (non emetic)	F837/76 (DSM 4222)	clinical isolate
B. cereus (non emetic)	HER 1399	environment
B. cereus (non emetic)	MHI 124	food
B. cereus (non emetic)	MHI 203	food
B. cereus (non emetic)	NVH 0154-01	food borne outbreak
B. cereus (non emetic)	NVH 0784-00	food borne outbreak

Table S1. continued

B. cereus (non emetic)	NVH 1104-98	food borne outbreak
B. cereus (non emetic)	NVH 1230-88	food borne outbreak
B. cereus (non emetic)	NVH 1651-00	food borne outbreak
B. cereus (non emetic)	NVH 449	food
B. cereus (non emetic)	NVH 506	food
B. cereus (non emetic)	NVH 655	environment
B. cereus (non emetic)	RIVM 485	food
B. cereus (non emetic)	RIVM 934	food
B. cereus (non emetic)	RIVM 938	food
B. cereus (non emetic)	SDA 1R 177	environment
B. cereus (non emetic)	SDA 1R 183	environment
B. cereus (non emetic)	SDA GR 281	food
B. cereus (non emetic)	SDA MA 57	food
B. cereus (non emetic)	SDA NFFE 664	environment
B. cereus (non emetic)	UHDAM B154	food borne outbreak
B. cereus (non emetic)	UHDAM TSP9	environment
B. cereus (non emetic)	WSBC 10286	food
B. cereus (non emetic)	WSBC 10441	environment
B. cereus (non emetic)	WSBC 10466	food isolate
B. cereus (non emetic)	WSBC 10483	food
B. cereus (non emetic)	WSBC 10885	clinical isolate
B. cereus^{T} (non emetic)	ATCC 14579 (DSM 31)	environment
B. cytotoxicus	CH_10	food
B. cytotoxicus	CH_106	food
B. cytotoxicus	CH_240	food
B. cytotoxicus	CH_246	food
B. cytotoxicus	CH_25	food
B. cytotoxicus	CH_29	food
B. cytotoxicus	CH_35	food
B. cytotoxicus	CH_38	food
B. cytotoxicus	CH_39	food
B. cytotoxicus	CH_73	food
B. cytotoxicus^{T}	NVH 0391-98	food borne outbreak
B. mycoides	BmA2	environment
B. mycoides	BmA3	environment
B. mycoides	BmCM1	environment
B. mycoides	BmFL2	environment
B. mycoides	BmFL3	environment
B. mycoides	BmLC1	environment
B. mycoides	WS 10256	environment
B. mycoides	WS 10257	environment
B. mycoides	WS 10258	environment
B. mycoides	WS 10277	food

Table S1. continued

B. mycoides	WS 10293	environment
B. mycoides	WS 10360	environment
B. mycoides	WSBC 10264	environment
B. mycoides	WSBC 10361	environment
B. mycoides^T	ATCC 6462	environment
B. thuringiensis	1/29 AGES	food borne outbreak
B. thuringiensis	2/27/S	food borne outbreak
B. thuringiensis	3/22 AGES	food borne outbreak
B. thuringiensis	6/27/S	food borne outbreak
B. thuringiensis	ATCC 29730	insect
B. thuringiensis	CH_10	food
B. thuringiensis	CH_130	insecticide
B. thuringiensis	CH_160	food
B. thuringiensis	CH_164	insecticide
B. thuringiensis	CH_181	insecticide
B. thuringiensis	CH_183	insecticide
B. thuringiensis	CH_185	insecticide
B. thuringiensis	CH_186	insecticide
B. thuringiensis	CH_187	insecticide
B. thuringiensis	CH_19	food
B. thuringiensis	CH_24	food
B. thuringiensis	CH_26	food
B. thuringiensis	CH_34	food
B. thuringiensis	CH_35	food
B. thuringiensis	CH_40	food
B. thuringiensis	CH_41	food
B. thuringiensis	CH_42	food
B. thuringiensis	CH_43	food
B. thuringiensis	CH_44	food
B. thuringiensis	CH_48	food
B. thuringiensis	CH_50	food
B. thuringiensis	CH_65	food
B. thuringiensis	CH_66	food
B. thuringiensis	CH_69	food
B. thuringiensis	CH_72	food
B. thuringiensis	CH_81	food
B. thuringiensis	CH_9	food
B. thuringiensis	CH_95	food
B. thuringiensis	CH_96	food
B. thuringiensis	CVUAS 2492	food borne outbreak
B. thuringiensis	CVUAS 9659	food borne outbreak
B. thuringiensis	CVUAS 9660	food borne outbreak
B. thuringiensis	HER 1211	environment

Table S1. continued

B. thuringiensis	HER 1231	environment
B. thuringiensis	HER 1232	environment
B. thuringiensis	HER 1236	environment
B. thuringiensis	HER 1357	environment
B. thuringiensis	HER 1387	environment
B. thuringiensis	HER 1418	environment
B. thuringiensis	P01_1	food
B. thuringiensis	P01_3	food
B. thuringiensis	P05_1	insecticide
B. thuringiensis	P05_2	insecticide
B. thuringiensis	PO1_Lonc	environment
B. thuringiensis	T05001	environment
B. thuringiensis	WS 2621	environment
B. thuringiensis	WS 2623	insect
B. thuringiensis	WS 2632	environment
B. thuringiensis	WSBC 28001	environment
B. thuringiensis	WSBC 28002	environment
B. thuringiensis	WSBC 28020	environment
B. thuringiensis	WSBC 28022	environment
B. thuringiensis	WSBC 28023	environment
B. thuringiensis	WSBC 28024	environment
B. thuringiensis^{T}	ATCC 10792	animal
B. weihenstephanensis	INRA 5	food
B. weihenstephanensis	INRA I20	food
B. weihenstephanensis	INRA I3	food
B. weihenstephanensis	SDA NFFE 647	environment
B. weihenstephanensis	WSBC 10067	food
B. weihenstephanensis	WSBC 10201	food
B. weihenstephanensis	WSBC 10202	food
B. weihenstephanensis	WSBC 10295	environment
B. weihenstephanensis	WSBC 10363	environment
B. weihenstephanensis	WSBC 10365	environment
B. weihenstephanensis	WSBC 10377	food
B. weihenstephanensis	WSBC 10378	food
B. weihenstephanensis	WSBC 10379	food
B. weihenstephanensis^{T}	DSM 11821 (WSBC10204)	food

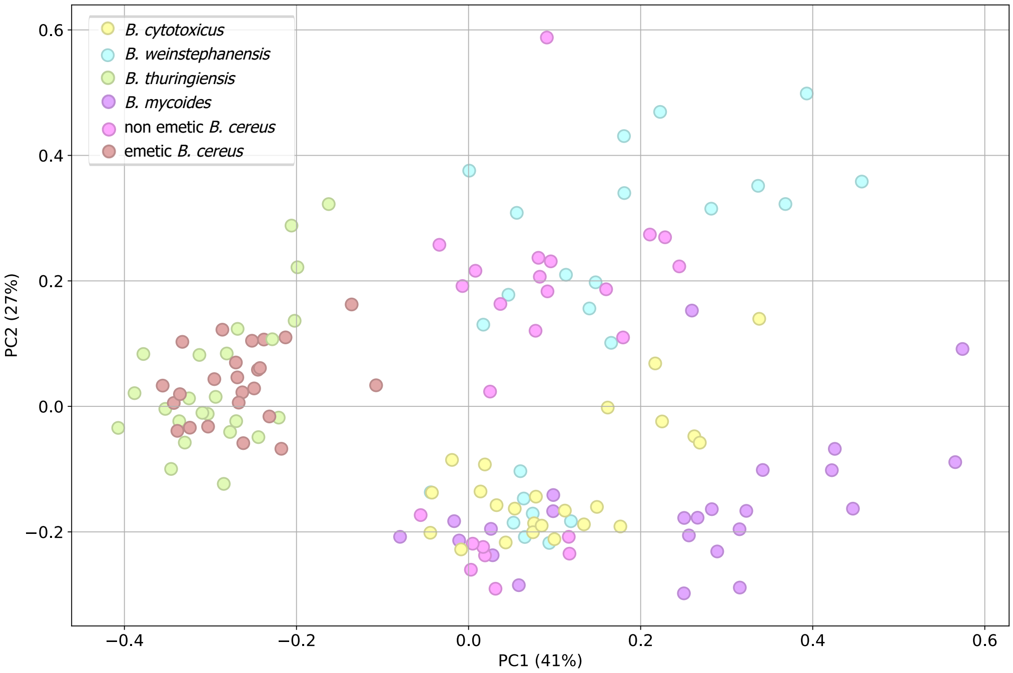

Figure S1. Principle component analysis (PCA) of pre-processed FTIR spectral data from *B. cereus* group species. Score plots of the PCA analysis are depicted. The percentages of the principal components are 41, 27, 12, 9, 6, respectively.

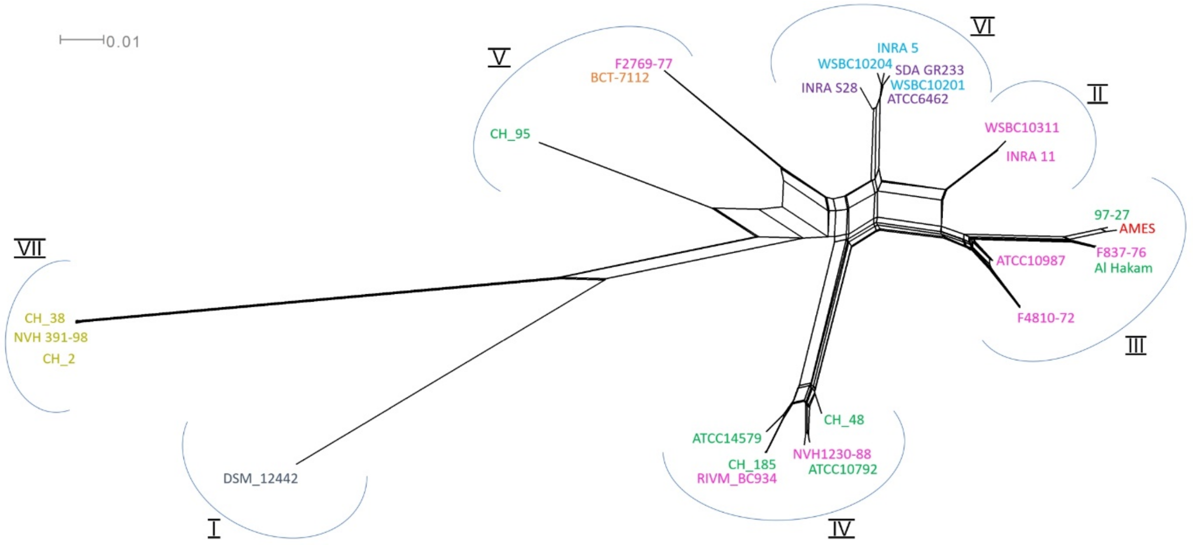

Figure S2. SplitsTree depicting the degree of similarity of the *panC* sequences from *B. cereus* group members. Strains are assigned to the seven *panC* types as defined by Guinebretiere *et al.*, (Guinebretiere *et al.*, 2008). The different species are indicated as followed: red: *Bacillus anthracis*; pink: *Bacillus cereus*, yellow: *Bacillus cytotoxicus*; purple: *Bacillus mycoides*; black: *Bacillus pseudomycoides*; green: *Bacillus thuringiensis*; orange: *Bacillus toyonensis*; blue: *Bacillus weihenstephanensis*

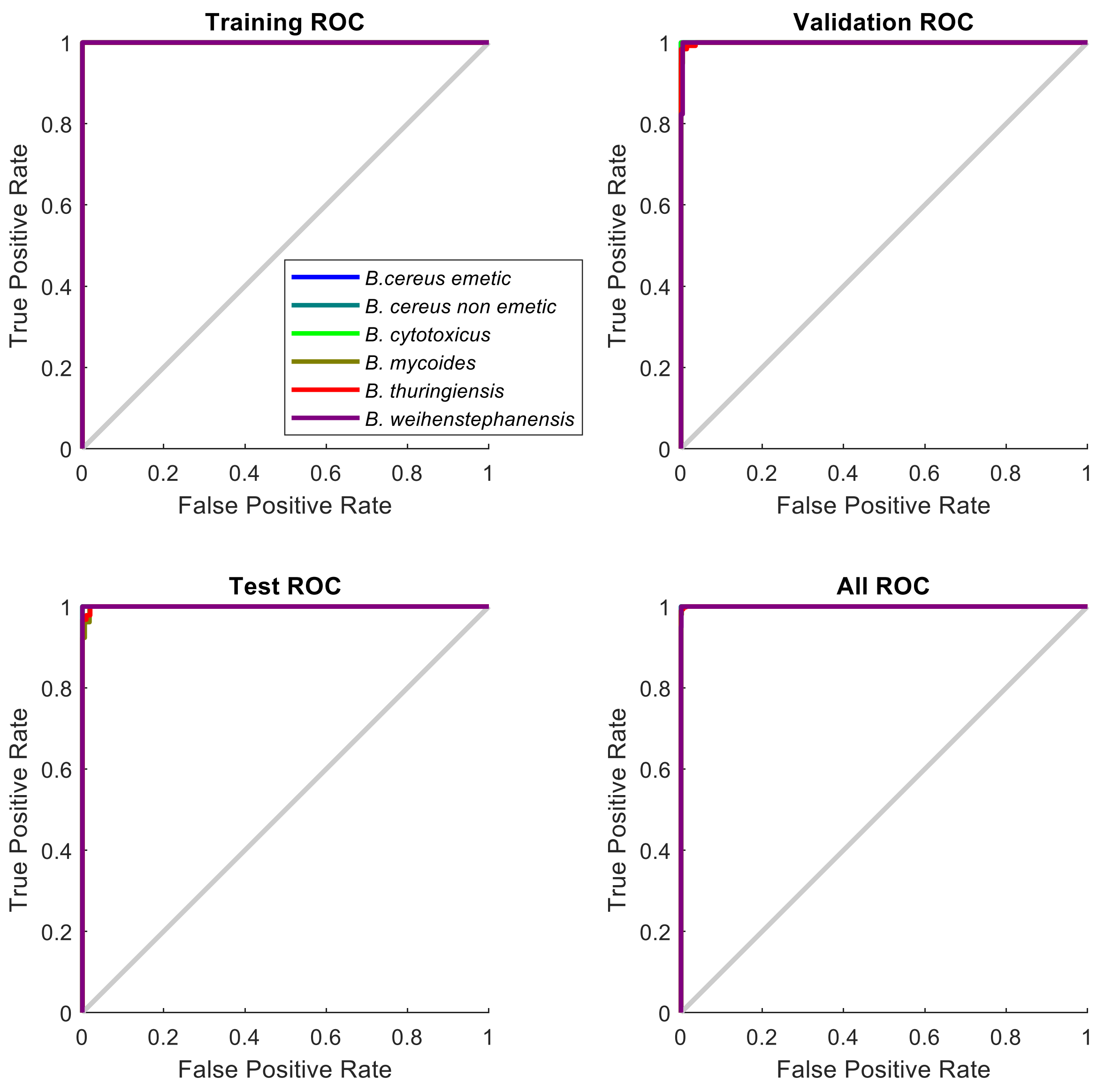

Figure S3. Receiver operating characteristics (ROC) curve analysis obtained from ANN classification method based on FTIR spectroscopic data. Successful model shows values close to 1.
